# Unmasking the reactants inhomogeneity in gas diffusion layer and the performances of PEMFC induced by assembly pressure

**DOI:** 10.1016/j.heliyon.2024.e32501

**Published:** 2024-06-06

**Authors:** Liu Yang, Z.Y. Sun, Guang-Meng Zhang, Zeng-Shan Li, Ke-Xuan Ren

**Affiliations:** a Hydrogen Energy and Space Propulsion Laboratory (HESPL), School of Mechanical, Electronic and Control Engineering, Beijing Jiaotong University, Beijing, 100044, China; b Beijing Long March Tianmin High-Tech Co., Ltd., Beijing, 100076, China; c Beijing Institute of Space Launch Technology, Beijing, 100076, China

**Keywords:** PEMFC, Assembly pressure effects, Gas diffusion layer, Transport properties, Dynamic performance

## Abstract

The gas diffusion layer (GDL), as the bridge to reactants and electrons in PEMFC, is a carbon-based porous component and would be deformed under compression to induce changes in the distributions of reactants and the corresponding performances of PEMFC; therefore, unmasking the impacts of assembly pressure on the distribution of the reactants in GDL is significant to improve the performance of PEMFC. In the present article, the structural response of GDL to assembly pressure was first studied; the variations in transport properties of GDL and the reactant distributions induced by assembly pressure were then discussed; the impacts on the dynamic performances of PEMFC were finally investigated. From the results, assembly pressure was found to have different effects on the regions of GDL under the rib and channel, significant gaps in GDL porosity and/or GDL permeability existed near the rib/channel transition region to worsen the inhomogeneity of reactants. Suffering assembly pressure, the distribution of current density became uneven, and the current density near the rib-channel border seriously rose to the aggravated risk of MEA thermal damage. Furthermore, the power density at specific efficiencies was raised under certain assembly pressures, which meant suitable assembly pressure(s) existed for better output performances of PEMFC.

## 1 Introduction

Hydrogen energy has received widespread acceptance for its substantial contribution to the future energy system since it is carbon-free and renewable [[1], [2], [3]]. The proton exchange membrane fuel cell (PEMFC), a classy variety of hydrogen fuel cells that operate at low temperatures and with remarkable efficiency [4,5], has gained increasing attention [6]. The actual open-circuit voltage of a single PEMFC is approximately 1 V (lower than the theoretical voltage of 1.23 V provided by the Gibbs free energy of hydrogen) for the polarization effects, PEMFCs assembled to form fuel cell stacks to meet the requirements of various practical applications. After being assembled, the practical performance of PEMFC would vary from its intended use, and these variations are thought to be related to assembly pressure [7]. To support the practical and substantial uses of PEMFC, fundamental research on the effects of assembly pressure on PEMFC has begun to raise scientific interest.

In the past decades, considerable outstanding studies have been done to examine the roles of assembly pressure on PEMFC. Lee et al. [8] and Wen et al. [9] respectively measured the voltage-current density curves (also known as polarization curves in literature) of single PEMFCs (with different gas diffusion layers, GDL) and PEMFC stack (with different bolt configurations), the findings showed that the assembly pressure would significantly affect the performances of PEMFC. Lee et al. [10] simulated (by the method of finite element analysis) the deformation process in PEMFC, and they reported that assembly pressure remarkably affects the pressure distribution on GDL. Zhao et al. [11] studied the temperature distribution in a PEMFC stack under various bolt torques and revealed that compression would influence the distribution uniformity of temperature via the impacts on the microstructure of GDL.

GDL, as the transportation pathways of gases, water, heat, and electrons [[12], [13], [14]], is a wet-proofed and carbon-based porous component. A variation of the structure in GDL, like thickness [14], surface roughness [15], the coupling effects of pore structure to hydrophobicity [16,17], the structure and form of contact between GDL and the channel of plate [18], and/or the detailed structure of microporous layers [19]) would induce remarkable variations of reactants transport and consequently the performances of PEMFC (the similar situation is also found in PEM electrolyzer [20]). Compared to the non-porous PEM and bipolar plates, the porous structure makes GDL have a lower Young's modulus, which makes the aforementioned structural parameters of GDL more susceptible to assembly pressure.

As Gostick et al. [21] (experiments) and Su et al. [22] (simulation) reported, compression force on the GDL seriously varied the porosity and permeability of GDL. In the optical experiments conducted by Kandlikar et al. [23], the flow within GDL was found maldistributed via the uneven GDL intrusion as the effect of compression force. Taymaz and Benli [24] simulated the assembly pressure effects on a single-straight channel PEMFC and reported increasing assembly pressure would weaken the performances of PEMFC for the declined porosity of GDL. In the measurements on the ohmic resistance of GDL conducted by Mason et al. [25], assembly pressure was considered to compress the thickness of GDL and resulted in a decline of ohmic resistance. Albeit PEM and CL also suffer the effects of assembly pressure [25,26], the major deformation induced by compression has been found to occur at GDL [27]. Therefore, the nexus between GDL deformation (like variations of thickness, porosity, permeability, etc.) and PEMFC performances has become a hot topic in the studies about assembly pressure effects on PEMFC in the current decade.

Heretofore, Zhou et al. [28] simulated the working process of a PEMFC with the type of single straight channel and reported that water distribution in GDL would be influenced by assembly pressure with nonmonotonic regulations. Cha et al. [29] and Tanaka et al. [30] studied the performances of PEMFC (respectively, with the type of single serpentine channel [29] and parallel channel [30]) and indicated assembly force would monotonously reduce ohmic resistance but the effects on voltage are nonmonotonic. Toghyani et al. [31] numerically studied the assembly pressure effects on PEMFC (with the single straight channel) and showed that assembly pressure could shorten the route of gas transportation by reducing GDL thickness (which brought positive effects) but reduced the transfer rate of gas by the reduced porosity (which bring negative effects). Movahedi et al. [32] reported increasing assembly pressure could lead to the drying of the membrane (to the decline of water content) which resulted in the worsening of PEMFC performances. Yan et al. [33] numerically studied the assembly pressure effects on a PEMFC stack (with the type of parallel channel) and indicated the assembly pressure would have essential impacts on the output power, which was like the findings by Ma et al. [34] upon the simulation of a single PEMFC (with the single straight channel). Son et al. [35] numerically studied assembly pressure effects on PEMFC with different types of channels (single serpentine, parallel, and interdigitated) and indicated the assembly pressure effects would be dependent on channel type and the crucial influence aspect to output performance is related to current density under a specific type of channel. In the recent simulation conducted by Moslemi et al. [36], the water withdrawal in PEMFC (with the interdigitated channel) was regarded as being significantly influenced by the level of compression on the fuel cell.

Upon the literature review, it could be expected that designing reasonable and suitable assembly pressure is significant to PEMFC, which should depend on clear and systematic cognitions of the nexus among assembly pressure, GDL deformation, and transportation of substances within PEMFC. In the reported works concerned assembly pressure effects, some works focused on the nexus between assembly pressure and the variations of GDL properties, and others mainly put the interest on the establishment of novelty models [[35], [36], [37]].

In the present work, a PEMFC with the channel type of single-straight was taken as the prototype to study the effects of assembly pressure. First, the effects of assembly pressure on the structure characteristics of GDL (indicated by interior stress, thickness, and porosity) were numerically investigated. Next, the transmission characteristics of GDL (indicated by permeability, oxygen distribution, and membrane water contents) were analysed and discussed. Then, the effects of assembly pressure on the performances of PEMFC (indicated by current density distribution, polarization curves, and power density) were compared. The main contribution of this article included: (a) the impacts of assembly pressure on the transmission characteristics within GDL were systematically studied, and (b) the nexus among assembly pressure, power density, and fuel cell efficiency was analysed.

## 2 Methodology

### 2.1 Settings of the source terms for PEMFC

For PEMFC, in combination with its momentum characteristics, its momentum conservation equation could be written as

(1) $$\frac{\partial \left(\varepsilon \rho \vec{v}\right)}{\partial t}+\nabla \cdot \left(\varepsilon \rho \vec{v}\vec{v}\right)=-\varepsilon \nabla p+\nabla \cdot \left(\varepsilon \mu \nabla \vec{v}\right)+S_{v}$$

where *p* was pressure (in Pascal), *μ* was dynamic viscosity (in Pa·s), $\vec{v}$ was velocity vector (in m/s), and *S*_v_ was momentum source term. Since both GDL and CLs were porous structures, the convection term and diffusion term could be ignored, and the right end of the above equation could be simplified to retain only the momentum source term which could be simplified upon Darcy's law to

(2) $$S_{v,gdl/cl}=-\frac{\mu }{K_{gdl/cl}}\vec{v}$$

where *K*gdl/cl was the permeability of GDL towards CL.

Similarity, based on the porous characteristics of GDL and CLs, under the assumption of continuous medium, the mass conservation equation of each component in the domain could be written as

(3) $$\frac{\partial \left(\varepsilon \rho \right)}{\partial t}+\nabla \cdot \left(\varepsilon \rho \vec{v}\right)=S_{m}$$

where *ε* was the porosity of porous media, *ρ* was the fluid density (in kg/m^3^), and *S*_m_ was mass source term. For anode and cathode CLs, the mass source terms could be written as

$$S_{m,a}=S_{H_{2}}=-\frac{M_{H_{2}}}{2F}i_{a}$$

(4) $$S_{m,c}=S_{H_{2}O}+S_{O_{2}}=\frac{M_{H_{2}O}}{2F}i_{c}-\frac{M_{O_{2}}}{4F}i_{c}$$

where *M* was the molar mass of a specific component in CL (in g/mol), *F* was Faraday constant (96485C/mol), *i* was the exchange current density (in A/cm^2^), _a_ and _c_ were respectively indicated anode and cathode.

In PEMFC, the energy was always conserved during operation with the total conservation equation as

(5) $$\frac{\partial \left(\varepsilon {\rho C}_{p}T\right)}{\partial t}+\nabla \cdot \left(\varepsilon {\rho C}_{p}\vec{v}T\right)=\nabla \cdot \left(k^{eff}\nabla T\right)+S_{Q}$$

where *C*_p_ was specific heat at constant pressure (in J/(kg·K)), *T* was the temperature (in K), *k*^eff^ was effective thermal conductivity (in W/(m·K)), and *S*_Q_ was energy source term. For PEMFC, the consumed energy involved reaction heat, resistance heat, phase change heat, and overpotential heat; thus, the energy source term could be written as

(6) $$S_{Q}={\left(i^{s}\right)}^{2}R_{\text{ohm}\;}+\beta S_{H_{2}O}h_{\text{reaction}\;}+r_{w}h_{\mathrm{lg}}+i_{a,c}\eta$$

where *i*^S^ was surface current density (in A/cm^2^), *R*_ohm_ was resistivity (in Ω·m), *β* was conversion rate which reflected ratio between the entropy term (T*△S) and the enthalpy (*h*_reaction_) term, *h*_reaction_ was the enthalpy of reaction (in J/mol), *h*_lg_ was the enthalpy of phase change from liquid water to gaseous (in J/mol), *r*_w_ was the flux of aqueous phase (in mol/s), and *η* was overpotential (in V).

The energy conversion was realized by the electrochemical reaction of hydrogen and oxygen, the simulation of PEMFC involved multi-component reaction flow, and the component conservation equation should be followed. In the model, the conservation equation of components could be written as

(7) $$\frac{\partial \left(\varepsilon c_{k}\right)}{\partial t}+\nabla \cdot \left(\varepsilon \vec{v}c_{k}\right)=\nabla \cdot \left(D_{k}^{eff}\nabla c_{k}\right)+S_{k}$$

where *C*_k_ was the concentration of each component (in mol/m^3^), *S*_k_ was the component source term, $D_{k}^{eff}$ was the effective diffusion coefficient of each component and determined by Bruggeman equation as

(8) $$D_{k}^{eff}=\varepsilon^{1.5}D_{k}$$

where *D*_k_ was the diffusion coefficient of binary components. The component source terms of hydrogen, oxygen and water vapour in the anode and cathode side catalytic electrodes could be expressed as

$$S_{k,a}=S_{H_{2}}=-\frac{M_{H_{2}}}{2F}i_{a}$$

(9) $$S_{k,c}=S_{H_{2}O}+S_{O_{2}}=\frac{M_{H_{2}O}}{2F}i_{c}-\frac{M_{O_{2}}}{4F}i_{c}$$

As a circuit system, the PEMFC model also comply with the charge conservation with the following equations as

$$\nabla \cdot \left(\sigma_{s}\nabla \varphi_{s}\right)+S_{s}=0$$

(10) $$\nabla \cdot \left(\sigma_{m}\nabla \varphi_{m}\right)+S_{m}=0$$

where *σ* was conductivity (in S/m), *φ* was potential (in V), *S*_s_ and *S*_m_ were respectively the potential source terms of solid phase electrons and membrane phase ions.

The present work fully considered the application forms of conservation equations in the constant-temperature PEMFC model and gave reasonable sets on the mentioned source terms (as listed in Table 1).

**Table 1:** Source terms of conservation equations.

Source Term	Symbol	Channel	GDL	CL (Anode)	CL (Cathode)	Membrane
Component Source Term	*S*_k_	0	0	*S*_H2_	*S*_O2_+*S*_H2O_	0
Electrochemical	*S*_s_	0	0	-*i*_a_<0	+*i*_c_ > 0	0
	*S*_m_	0	0	+*i*_a_ > 0	-*i*_c_<0	0
Mass	*S*_m_	0	0	*S*_H2_	*S*_O2_+*S*_H2O_	0
Momentum	*S*_v_	0	*S*_v,gdl_	*S*_v,cl_	*S*_v,cl_	0

### 2.2 Model setups of elastodynamics

As the bridge for the reactants between CL and the flow field plate, the structure of GDL requires porous type and the material of GDL is mainly carbon fibre [15,16] to reduce the contact resistance with the flow field plate (often made of non-porous carbon) and CL (often made of Pt/C). In an actual GDL, carbon fibres are randomly and horizontally stacked and bound into a network-like matrix by a polymeric binder [19], which results in different GDLs and different localizations on the same GDL having unique architecture. However, from the viewpoint of macroscopic deformation with mathematical statistical significance, the porous carbon fibre components have good linear elasticity, and the structural response (like displacement, stress and strain) under assembly pressure could be simulated and analysed by linear elastic mechanics.

**Fig. 1:**
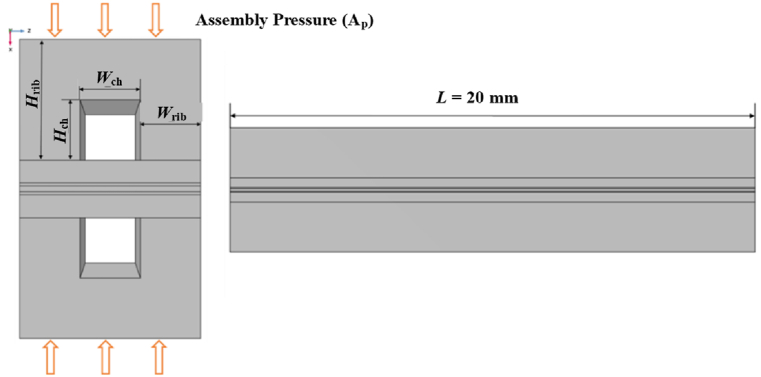
Computational domain of the solid mechanics model.

In the present study, the computational domain was a single straight channel, which is the most basic configuration and may potentially be thought of as the microelement structure of any channel type. In the solid mechanics module solution, the assembly pressures acting on the flow field plates are equally high and evenly dispersed, as demonstrated in Fig. 1, while the main parameters of the geometric model have been listed in Table 2.

(a) the assembly pressure acting on the bipolar plates on both sides was equal and uniformly distributed.
(b) the structures of GDL and CL were continuous.
(c) the materials of GDL and CL were elastic, and their stress and strain relationships conformed to Hooke's law.
(d) the mechanical properties of GDL and CL (like Poisson's ratio, modulus of elasticity and density) were uniform and isotropic.
(e) the displacements of GDL caused by the assembly pressure were minute.
(f) the initial stress of each component in the PEMFC was zero.

**Table 2:** Main parameters of the geometric model.

Parameters	Height (along X-axis)	Long (along Y-axis)	Width (along Z-axis)	Young's modulus (MPa)	Poisson's ratio	Density (kg/m^3^)	Conductivity (S/m)	Initial Porosity
GDL	0.38 mm	20 mm	3 mm	5.54	0.256	1000	1200	0.78
CL	0.05 mm	20 mm	3 mm	249	0.300	1980	222	0.40
Membrane	0.10 mm	20 mm	3 mm	232	0.253	1000	/	/
Channel	1.00 mm	20 mm	1 mm	13000	0.260	1000	20000	/
Rib	1.00 mm	20 mm	1 mm					

Based upon the theory of linear elastic mechanics, the following assumptions were made in the investigation as [38].

Furthermore, both sides of the bipolar plate were set as symmetric boundary conditions, the membrane was set as a fixed constraint, and the remaining faces were set as free surfaces.

### 2.3 Model setups of electrochemical and water transport

The relationship between the current and potential generated by the electrochemical reaction of the PEMFC was under the Butler-Vomer equation as

$$i_{a}=\zeta_{a}j_{a}^{ref}{\left(\frac{c_{H_{2}}}{c_{H_{2}}^{ref}}\right)}^{\gamma_{a}}\left[e^{\left(\frac{\alpha_{a}F\eta_{a}}{RT}\right)}-e^{\left(-\frac{\alpha_{c}F\eta_{a}}{RT}\right)}\right]$$

(11) $$i_{c}=\zeta_{c}j_{c}^{ref}{\left(\frac{c_{O_{2}}}{c_{O_{2}}^{ref}}\right)}^{\gamma_{c}}\left[e^{\left(\frac{\alpha_{a}F\eta_{c}}{RT}\right)}-e^{\left(-\frac{\alpha_{c}F\eta_{a_{c}}}{RT}\right)}\right]$$

where *ζ* was the active specific surface area (in 1/m), *j*^ref^ was the reference exchange current density (in A/cm^2^), *c*_i_ and *c*_i_^ref^ were the molar concentrations of each component and the corresponding reference concentration (in mol/m^3^), *γ* was the index of concentration, *α* was the transfer coefficient, *R* was the ideal gas constant, and *η* was the overpotential (namely the voltage loss).

Protons cannot be transferred between the catalytic electrode and the membrane's electrolyte unless they are in the hydrated state of the electrolyte. The degree of hydration of the electrolyte was indicated by the membrane state (*λ*) which was determined by the combination of the equivalent mass of the membrane (*E*w, in g/mol), the density of the membrane in the dry state (*ρ*_mem_, in g/m^3^), and the concentration of water in the electrolyte (*C*_H2O_, in mol/m^3^) as

(12) $$\lambda =\frac{EW}{\rho_{\text{mem}\;}}C_{H_{2}o}$$

Upon the Nernst-Plank law, water in an electrolyte consisted of the transmembrane flux of water due to the concentration difference (*J*_mw,diff_, namely, the difference in water concentration between the two electrodes), electro-osmotic drag effects (*J*_mw,eod_, namely, the certain number of water molecules dragged by the protons from the anode to the cathode through the process of the membrane), and the hydraulic osmosis (*J*_mw,hyd_, namely, the difference in pressure between the two electrodes). Learnt from Springer et al. [39], the transmembrane flux of water due to hydraulic osmosis was unremarkable, and it was ignored in the present investigation. The amounts of the water transport were controlled by the diffusion rate of water (*D*_mw_, in m^2^/s) and the electro-osmosis drag coefficient (*n*_d_) as

$$J_{mw,diff}=-D_{mw}\nabla c_{mw}=-\frac{\rho_{m}}{EW}D_{mw}\nabla \lambda$$

$$J_{\text{mw},\text{eod}\;}=n_{d}\frac{I_{H^{+}}}{F}$$

(13) $$J_{mw,diff}=-D_{mw}\nabla c_{mw}=-\frac{\rho_{m}}{EW}D_{mw}\nabla \lambda J_{\text{mw},\text{eod}\;}=n_{d}\frac{I_{H^{+}}}{F}$$

Based upon the mentioned governing equations, the following assumptions were made in the investigation as [40]:

(a) the flow of the reactants was regarded as laminar.
(b) the reactants were regarded as ideal gases while gravity was neglected.
(c) the porous structures were modelled with a macro-homogeneous approach, characterized by uniform porosity and permeability.
(d) the PEM was impermeable to gaseous species.

### 2.4 Investigation of the mesh independence

Considering the structure of PEMFC was relatively regular and lamellar, the strategy of hexahedral mesh was adopted to ensure high mesh quality. Upon the structured hexahedral mesh, a validation study of mesh independence was carried out. By controlling the maximum size of the mapped grid in the whole domain of the computational model to be 2.5e^−4^ m, and gradually increasing the number of layers of the membrane, CL, GDL, and other components of the encryption method, it was ensured that the average and the lowest quality of the six meshing schemes were the same. Detailed settings were listed in Table 3. All the simulations in the present work were conducted by COMSOL Multiphysics.

**Table 3:** Mesh refinement method and the obtained current density under the operation voltage of 0.7 V.

	Layer number of each component				Mesh number	Current density (A/cm^2^)
	GDL	CL	Membrane	Channel/Rib		
Case I	3	1	1	3	115,200	0.556
Case II	3	2	2	3	144,000	0.517
Case III	4	3	2	4	192,000	0.501
Case IV	4	3	3	4	201,600	0.501
Case V	4	3	3	5	211,200	0.502
Case VI	5	3	3	5	230,400	0.513

**Fig. 2:**
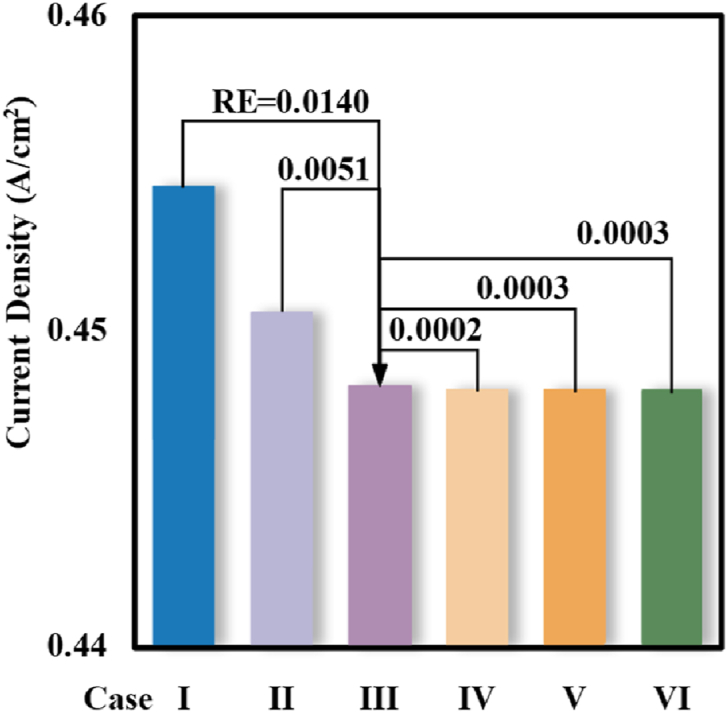
Obtained current density at 0.7 V and the related error upon Case III in the simulation with different mesh strategies.

Fig. 2 compared the current densities and the corresponding relative errors of the six meshing strategies under a typical operation voltage (0.7 V). As could be observed, upon the simulation result obtained in Case III, the relative errors of the current density were tiny albeit the mesh was further refined. Therefore, the settings in the Case III strategy were adopted in the present. Furthermore, the simulation results had been validated by the experimental data, and more information could be found in previous literature [5].

## 3 Results and discussion

### 3.1 Brief statements about the impacts of assumptions on the actual results

As mentioned above, the simulations were conducted with some assumptions; therefore, it is necessary to have brief statements on the impacts of assumptions on the actual results before systematic discussions on the numerical results.

The major differences between the assumptions and the actual were the considerations of isotropic and uniform properties of GDL, and another remarkable difference was the equal and uniform distribution of assembly pressure as the initial condition. The actual microstructure of GDL is complex and anisotropic, which determines the effective properties of a specified GDL challenging [12]; the pressure distribution is related to assembly manner [41], and the uniformity degree would induce the detailed microstructure of GDL more complex under different assembly pressure. Since the microstructure of GDL is random, the detailed variations of microstructure would be more complex under non-uniform compression for the coupled impacts which made it difficult to qualitatively analyze the nexus between assembly pressure and different parameters; therefore, the present study had to simplify the studied cases with the assumptions.

Meanwhile, the available works highlighted the variations of GDL thickness under compression would play a more remarkable role in the changes in fuel cell's performances [42] for the varied porosity [43] and electrical resistance [44], and the nexus between assembly pressure and such variants could be obtained with the mentioned assumptions for mathematical statistical significances. Furthermore, the available works proved the simulation results with the mentioned assumptions could reflect the actual variation regulations with the comparisons of experimental results [[45], [46], [47]].

Upon the mentioned, albeit the obtained results were idealized, on whom the discussions were still valuable to unmasking the impacts of assembly pressure on the reactants distribution and the corresponding performances of PEMFC.

### 3.2 Effects on the structure characteristics of GDL

The components' deformation by assembly pressure mostly affects GDL since Young's modulus of the other components is significantly higher than that of GDL. To evaluate assembly pressure effects on the structure of GDL, six groups of cases with assembly pressure (**A**_***p***_) of 0.5 MPa, 1.0 MPa, 1.5 MPa, 2.0 MPa, 2.5 MPa, and 3.0 MPa between each single cell were numerically simulated.

**Fig. 3:**
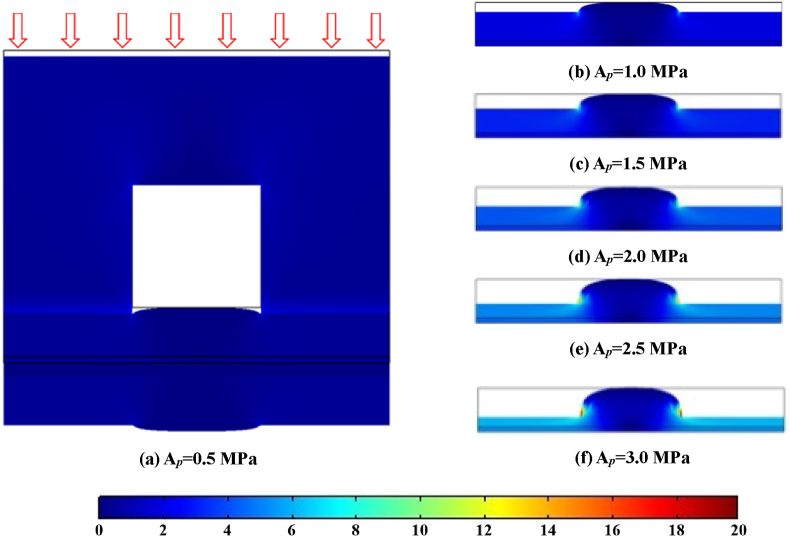
Deformation together with von Mises stress (in MPa) distribution in GDL under different assembly pressures.

Fig. 3 demonstrated the deformation of GDL together with the stress distribution under various assembly pressures. Suffering the effects of assembly pressure, the regions of GDL under the rib were compressed closer and more uniformly (as observed in the experiment [48]), and the degree of extrusion deformation (in the regions under the rib) gradually increased with the increase of assembly pressure. Meanwhile, to the regions of GDL under the channel, the space of the channel resulted in a preference for stress conduction along the interface of the channel to rib, which led to accumulated effects of stress concentration at the rib/channel interface and correspondingly a Hump-shape deformation of the GDL under the channel. With the increase of assembly pressure, the phenomenon of stress concentration at the rib edge became more noticeable, and the deformation of GDL under the rib became more serious.

**Fig. 4:**
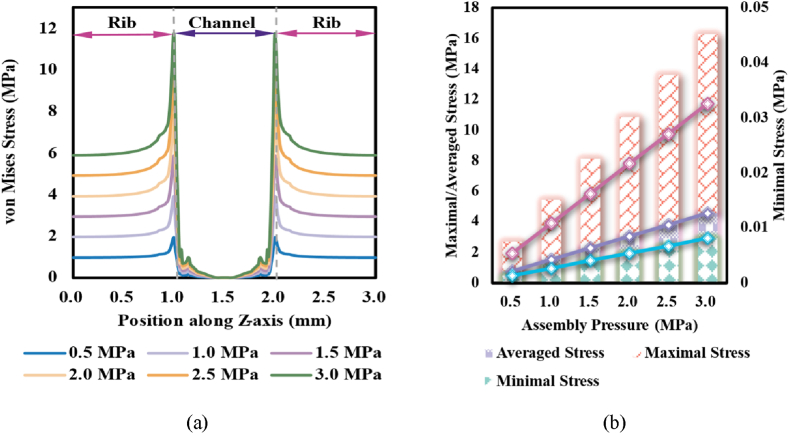
Stress on GDL: (a) von Mises stress distribution in GDL; and (b) averaged and extremal values of stress in GDL.

Fig. 4(a) demonstrated the distribution of von Mises stress on the cross-section of the GDL under various assembly pressures. Besides the remarkably raised stress on the GDL, with the increase of assembly pressure, the stress oscillation observed near the rib/channel interface became more intense. Such oscillation was considered the result of discontinuous transitions in the responses of GDL to compression. To the regions of GDL under the rib, the initially separated carbon fibres (far away from the rib/channel interface) would be compressed to occupy the original pore space [45], but the compressed carbon fibres (near the rib/channel interface) would bend to the space under the channel [31]; meanwhile, the effects of assembly pressure on the channel would be conducted along the junction of rib and channel, which resulted to the gradient impacts on the carbon fibres under the channel. As the compression intense rose, the accumulated effects of stress in the transition regions became stronger, and the differences in the deformations of GDL in the transition regions would be more obvious. From the comparison of the averaged and the extreme values of stress (Fig. 4 (b)), it could be observed that all the indices linearly rose with the increase of assembly pressure, and the peak stress was about 156 % of the averaged stress while 390 % of the assembly pressure.

**Fig. 5:**
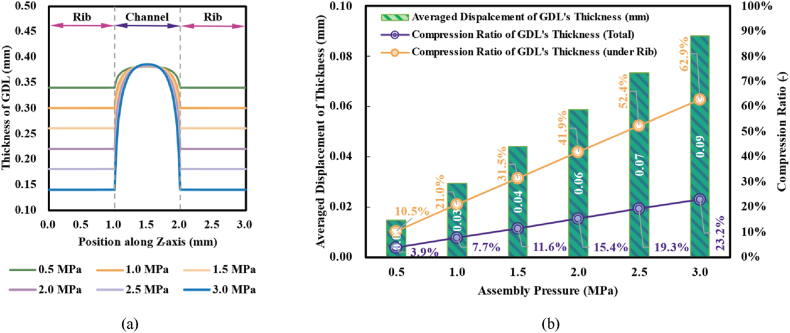
Variations of GDL's thickness under different assembly pressures: (a) distribution of thickness; and (b) indicating values of thickness displacement together with compression ratios.

Fig. 5 demonstrated detailed information about the deformation of GDL under various assembly pressures. As could be observed, albeit the thickness of other partial GDL remarkably declined, the thickness of GDL at the central position (of the regions under the channel, namely the top position of the Humpback) was raised to occupy the channel space Undergoing 0.5 MPa, the compression ratio (defined as the ratio of averaged displacement undergoing assembly pressure to initial thickness in a free state) of the total thickness was about 3.9 % while the compression ratio of the regions under rib was about 10.5 %; as the assembly pressure rose to 3.0 MPa, the compression ratios respectively rose to about 23.2 % and 62.9 %. The significant gap of thickness between GDL under the rib and the regions under the channel would cause uneven transport properties and current density, which would seriously influence the performance of PEMFC.

### 3.3 Effects on the transmission characteristics of GDL

As a porous structure with compressibility, the thickness of GDL varied would induce variations of porosity and permeability, which led to changes in transport resistance and the flow velocity of reactant gases, drastically influencing the performances of PEMFC.

**Fig. 6:**
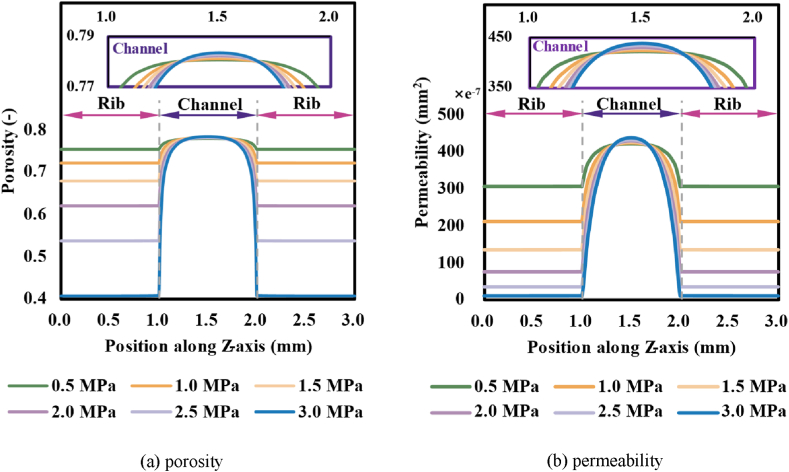
Distributions of porosity and permeability in the GDL under different assembly pressures.

Fig. 6 demonstrated the distributions of porosity and permeability of GDL under various assembly pressures. As could be observed, the porosity of GDL under the ribs on both sides significantly declined as assembly pressure rose, which was similar to the variations of GDL's thickness caused by assembly pressure since the porous space had been reduced by lateral compression, the decline extent reached to about 47.9 % when the assembly pressure had been raised to 3 MPa. Meanwhile, as the result of the reversed deformation of GDL, the porosity of GDL at the centre of the channel expressed a slight growth as assembly pressure rose, which in turn reduced permeability and effective diffusivity and thus mass transfer rates. Permeability, as a factor directly related to transport space, expressed a remarkable decline under the rib with the increase of assembly pressure, albeit a tiny increase at the centre of the channel. Furthermore, the higher the assembly pressure, the lower the permeability in the rib edge region close to the flow channel, and the range of the region under the flow channel affected by the action of assembly pressure rose.

**Fig. 7:**
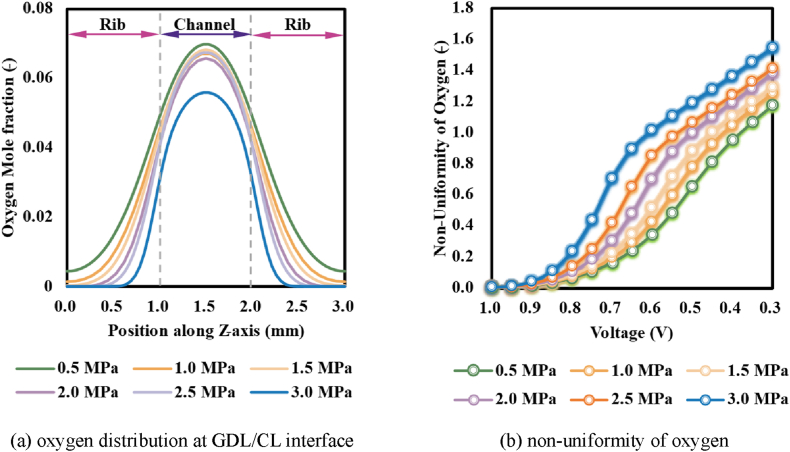
Distribution of oxygen and the uniformity of oxygen in the GDL under different assembly pressures.

Fig. 7 (a) demonstrated the oxygen distribution at the GDL/CL interfaces under different assembly pressures. As could be observed, oxygen was mainly concentrated in the region of GDL under the channel and the peak value of the oxygen mole fraction was attained at the centre of GDL. As the assembly pressure rose, the oxygen concentration remarkably declined due to the deterioration of mass transport induced by the gradual decrease in porosity after compression. When the assembly pressure was 3.0 MPa, the oxygen mole fraction at the centre of GDL (namely the peak value that could be obtained within the GDL) was seriously reduced by about 20 % (from that under 0.5 MPa). In contrast, the oxygen mole fraction had been reduced close to zero in a portion of the subcostal GDL where the range would be continuously enlarged as assembly pressure rose. This indicated assembly pressure would worsen oxygen transfer within GDL by reducing the permeability, and it also would exacerbate the uniformity of oxygen concentration in GDL between the subcoastal region and that under the channel (as demonstrated in Fig. 7 (b)) since the drop in GDL permeability under different regions had been enhanced.

With the decline of operation voltage, the electrochemical reaction had been accelerated and more reactants were required, the difference in the permeability of GDL under different regions would lead to higher non-uniformity, thus the inhomogeneity of oxygen rose [5] as could be observed from Fig. 7(b). With the increase of assembly pressure, the inhomogeneity of oxygen remarkably rose albeit the variations were unremarkable under higher voltage (like more than 0.9 V). When a PEMFC operated under higher voltage, due to the low rate of electrochemical reactions, the amounts of reactants were insufficient to saturate the permeability of GDL. With the decline of operation voltage (like to the range of 0.8 V–0.3 V, the current density rose, and more reactants were required for maintain the electrochemical reaction, the reduced GDL porosity in the subcostal region would significantly reduce the gas diffusion rate and thus the inhomogeneity of oxygen would be enhanced. The higher the assembly pressure was, the more serious deterioration of GDL porosity became, and the more remarkable non-uniformity of oxygen under lower voltage expressed.

**Fig. 8:**
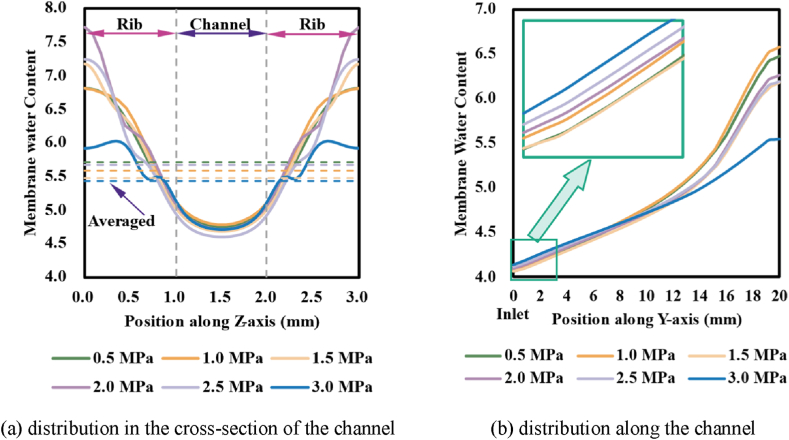
Distribution of membrane water content under different assembly pressures.

Membrane state water content is a direct indicator of the reaction and the level of flooding in the cell. Since the obstacle of rib, more water accumulated in the subcoastal region than under the channel region; meanwhile, the maximal gas flow rate was attained at the centre of the channel for better drainage, which resulted in a minimal value of water content at the centre of the channel and gradually rose towards the rib edges on both sides (as demonstrated in Fig. 8 (a)). As assembly pressure rose (0.5–3.0 MPa), the average water content expressed an overall decrease with rising assembly pressure, but it would rise when the assembly pressure was 2.0 MPa and/or 2.5 MPa. Raising assembly pressure reduced the oxygen concentration and consequently less water was produced, but the reduced permeability also reduced the drainage process, both two factors influenced the through-plane convection within the GDL, and thus the non-monotonic variations would appear. Furthermore, the planar convection along the channel appeared non-monotonic variation with the increase of assembly pressure (as demonstrated in Fig. 8 (b)) for the combined effects of permeability and the non-uniformity of oxygen induced by inhomogeneous compression of assembly pressure. The observed results indicated that assembly pressure plays a role in water management by inhomogeneous compression.

### 3.4 Effects on the dynamic performances of GDL

**Fig. 9:**
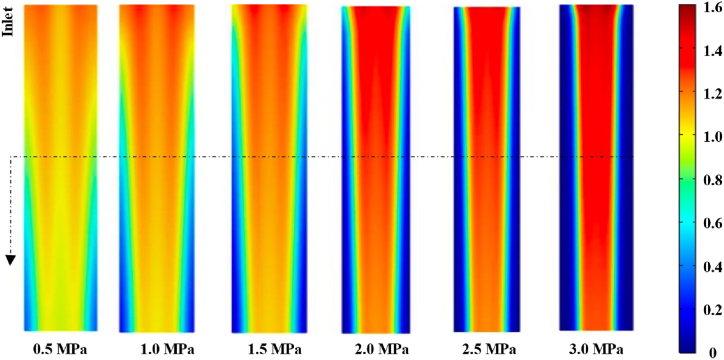
Current density at the centre interface of the membrane at 0.45 V.

Fig. 9 demonstrated the contour of current density at the central section of the membrane under an operation voltage of 0.4 V. The current density attained its maximal value at the inlet of gas flow and declined along the flow direction for oxygen consumption in the electrochemical reaction. As assembly pressure rose, the current density under the rib declined significantly while the current density under the channel rose instead; furthermore, the inhomogeneity of the current density was exacerbated. To observe and describe this phenomenon more clearly, the current density distribution was analysed by taking the transverse line at the centre.

**Fig. 10:**
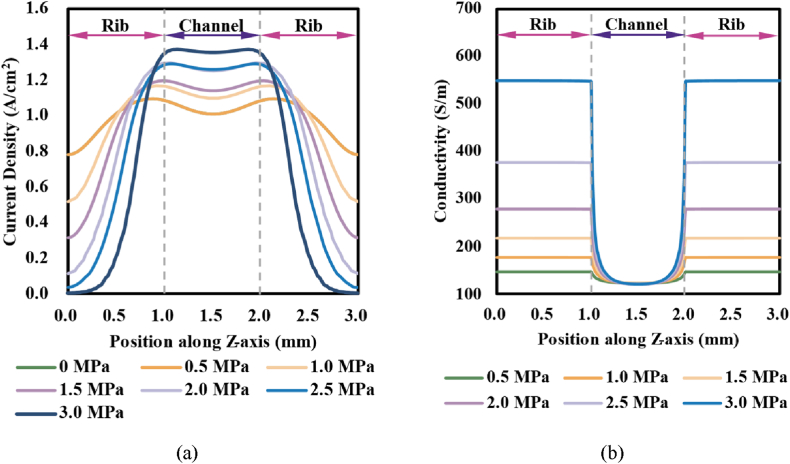
Distributions of current density (a) and GDL conductivity (b) under different assembly pressures.

As demonstrated in Fig. 10 (a), current density expressed concave distributions along the Z-axis, and it was greater close to the rib-channel border for the balance of mass transfer resistance in GDL and the electrical resistance in GDL material [21]. With the increase in assembly pressure, the GDL porosity under the rib was reduced while the porosity under the channel was raised, the transfer resistance was correspondingly varied, and thus the GDL conductivity under the rib rose while the conductivity under the channel declined (as demonstrated in Fig. 10), which facilitated the conduction of electrons through the ribs to the external circuitry. Therefore, as assembly pressure rose, the current density under the rib seriously declined while the current density under the channel remarkably rose, the maximal value of current density rose, and the corresponding position to the maximal current density became closer to the rib-channel border.

**Fig. 11:**
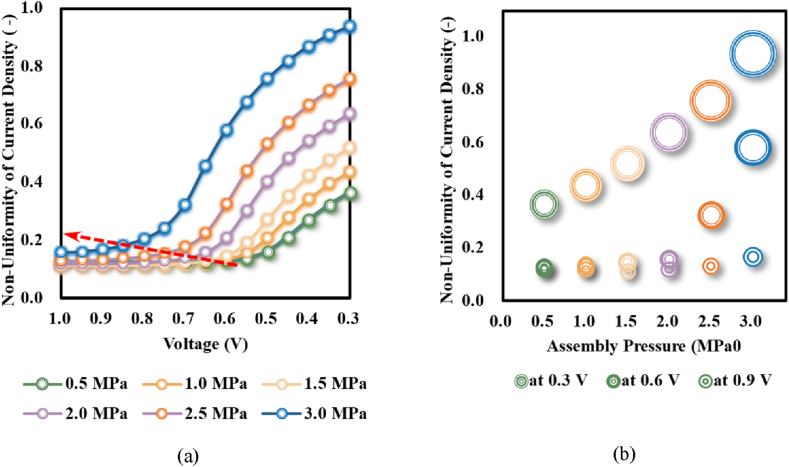
Non-uniformity of current density: (a) nexus to operation voltage; and (b) nexus to assemble pressure.

Furthermore, as assembly pressure rose, the uniformity of current density was seriously worsened and the variation extent significantly rose with the decline of operation voltage, as demonstrated in Fig. 11. The mentioned results indicated that a higher assembly pressure would not only induce hot spots near the rib-channel border but also worsen the distribution of current density (correspondingly the temperature), which would damage MEA and consequently PEMFC.

**Fig. 12:**
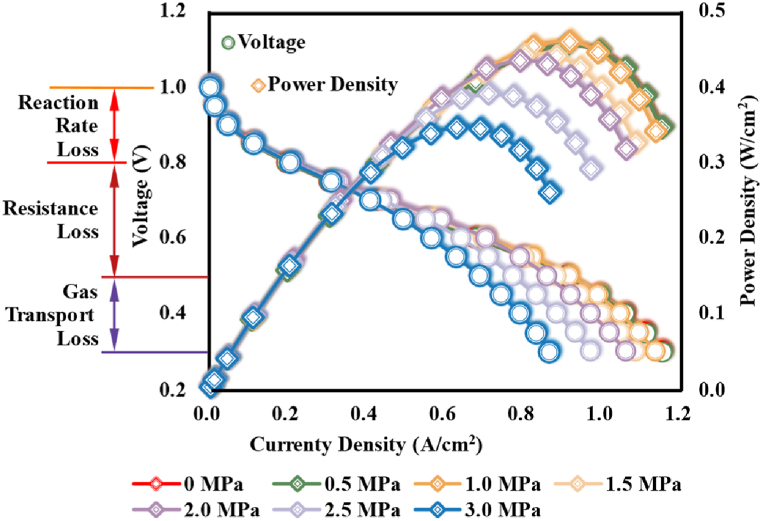
Dynamic performances of PEMFC under different assembly pressures.

As the demonstrated polarization curves in Fig. 12 (a), the impacts of assembly pressure were mainly felt in the region of Ohmic polarization (resistance loss) and the region of concentration polarization (gas transport loss). As assembly pressure rose, the generated current density under the same operation voltage would decline (outside the region of activation polarization). Albeit the contact area of the micro-contact points between the fibres through which the current past would be enhanced and the contact resistance would be reduced under compression effects [48], the remarkable decline of conductivity as mentioned above seriously enhanced the ohmic loss with the increase of assembly pressure. Meanwhile, the limited current density was controlled by the transport capability of reactants, which was determined by the porosity and the water content. As assembly pressure rose, the gas transport was seriously worsened and the oxygen distribution was more inhomogeneous, especially at high current loads (like between 0.6 and 1.2), which weakened the performances of PEMFC.

**Fig. 13:**
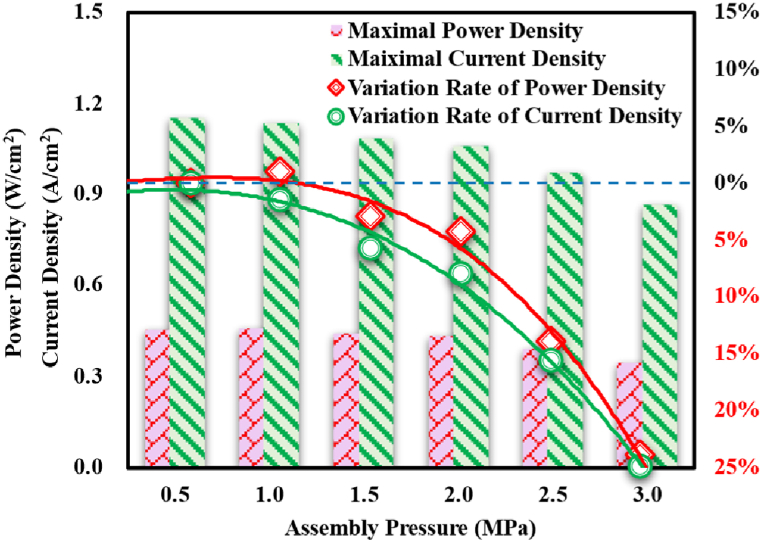
Maximal values and the variations rates of PEMFC's dynamic performances under different assembly pressures.

As determined by the voltage and current density, the power density of PEMFC would be reduced under higher assembly pressure. As could be observed, as assembly pressure rose, the maximal power density the PEMFC could obtain reduced albeit the reduction was unobvious under small assembly pressure (like no more than 1.0 MPa), and the current density to which the maximal power density corresponding was also reduced. Fig. 13 demonstrated the maximal values of power density and current density the studied PEMFC could obtain under different assembly pressures, together with the variations rates. As could be observed, suffering the compression of assembly pressure, an insufficient assembly pressure (like 0.5 MPa) hardly induces the change in dynamic performances of PEMFC; however, a suitable assembly pressure (like 1.0 MPa) could raise the maximal power density albeit the growth was not remarkable, which meant the performance would be enhanced a little. With assembly pressure further rising, the maximal dynamic performances of PEMFC would acceleratingly weaken.

**Fig. 14:**
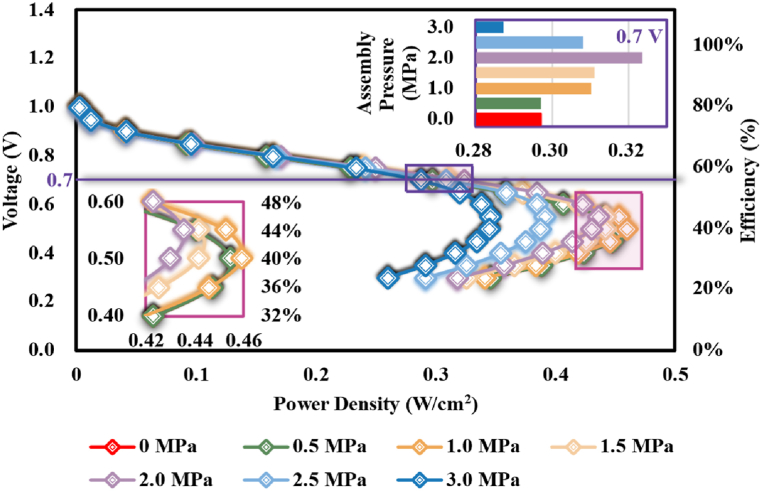
Nexus among power density, operation voltage, and PEMFC efficiency under different assembly pressures.

Fig. 14 demonstrated the nexus between power density and efficiency of the studied PEMFC under different assembly pressures. As could be observed, without assembly pressure, the studied PEMFC could obtain a maximal power density of approximately 0.455 W/cm^2^ at an efficiency of approximately 40 %. Insufficient compression (like 0.5 MPa) and suitable compression (like 1.0 MPa) hardly vary the corresponding efficiency to the maximal power density, but a higher assembly pressure would raise the efficiency at which the maximal power density would be obtained. It's worth noting that, compared to the power density at the voltage of 0.7 V (approximately 0.3 W/cm^2^ with an efficiency of approximately.

56 %) without compression, the assembly pressure of 2.0 MPa would raise the power density by approximately 9 % but a higher assembly pressure (like 3.0 MPa) would worsen the power capability, which also meant there existed a suitable assembly pressure for improving the output performance of PEMFC with different requirements on efficiency.

## 4 Conclusions

In the present article, the impacts of assembly pressure on structural response and transport properties of a GDL were first studied, and then the effects on the dynamic performances of PEMFC were simulated upon the computational domains with deformation geometries under different assembly pressures. The main conclusions were as follows:

(1) assembly pressure effects on the structure and transport properties of GDL mainly occurred in the region under the rib, in which the compressed GDL thickness resulted in the reductions of porosity and permeability; however, the partial GDL under the channel was pulled to form a Hump-shape for the higher stress on the rib-channel border.
(2) The assembly pressure induced significant gaps in the porosity and permeability of GDL between the regions under the rib and the channel, which caused uneven oxygen distributions, water content, and current density. The phenomena of flooding and the hot points at the rib-channel border would be worsened.
(3) Albeit the current density was overall reduced under the effects of assembly pressure for the enhanced resistance loss and gas transport loss, the power density at specific PEMFC efficiency was observed to rise under certain assembly pressures, which meant the existence of a suitable assembly pressure for the output performances of PEMFC.

## Data availability

Data will be made available on request.

## CRediT authorship contribution statement

**Liu Yang:** Writing – original draft, Validation, Software, Investigation, Formal analysis, Data curation. **Z.Y. Sun:** Writing – review & editing, Supervision, Resources, Methodology, Funding acquisition, Formal analysis, Conceptualization. **Guang-Meng Zhang:** Software, Formal analysis, Data curation. **Zeng-Shan Li:** Formal analysis, Data curation. **Ke-Xuan Ren:** Formal analysis.

## Declaration of competing interest

The authors declare that they have no known competing financial interests or personal relationships that could have appeared to influence the work reported in this paper.
